# Acute Flaccid Tetraparesis after COVID-19 Infection: Think of the Thyroid

**DOI:** 10.1155/2022/5827664

**Published:** 2022-04-29

**Authors:** Sarah Ying Tse Tan, Jiaqing Xiong, Troy H Puar, Joan Khoo, Andy Jun-wei Wong, Shui Boon Soh

**Affiliations:** Department of Endocrinology, Changi General Hospital, Singapore

## Abstract

A previously well 32-year-old Chinese male presented with acute bilateral upper and lower limb paralysis upon waking, ten days after the onset of COVID-19 infection. Examination revealed areflexia over all four limbs, associated with reduced muscle strength, but no sensory or cranial nerve deficit. Initial concern was Guillain-Barre syndrome given the acute flaccid paralysis following COVID-19 infection. However, investigations revealed severe hypokalaemia (1.7 mmol/L) and primary hyperthyroidism. He was treated for thyrotoxic periodic paralysis (TPP) with *β*-blockers, antithyroid medications, and intravenous potassium chloride (KCl). Despite frequent monitoring of potassium, rebound hyperkalaemia occurred with prompt resolution of paralysis.

## 1. Introduction

This is the first case report of TPP occurring after COVID-19 infection, which presented with acute flaccid tetraparesis. COVID-19 infection is associated with multiple complications including neurological, endocrinological, and autoimmune diseases. TPP is an uncommon complication of Graves' disease and may mimic other neurologic complications of COVID-19 infection such as demyelinating disorders. This emphasises the importance of assessing for metabolic disorders. In patients with severe hypokalaemia, a thyroid panel should be checked, bearing in mind the association between COVID-19 infection and Graves' disease. Finally, the acute management of patients with TPP and severe hypokalaemia can be challenging, and judicious potassium replacement with frequent monitoring is imperative to avoid rebound hyperkalaemia.

## 2. Case Summary

A previously well 32-year-old Chinese male presented to the emergency department with acute weakness of bilateral upper and lower limbs. He slept well the night before, but was unable to walk or lift his lower limbs upon waking. Upper limb strength was also reduced bilaterally. He had no numbness, facial drooping, slurring of speech, blurring of vision, or incontinence. There was no preceding headache, neck pain, or trauma. He had been diagnosed with COVID-19 upper respiratory tract infection ten days ago, and his symptoms of fever, runny nose, cough, and sore throat had since resolved. However, he reported weight loss of 5 kg over the past few weeks. He took a carbohydrate-rich diet mainly consisting of rice, which he had taken the night before as well. He did not notice any neck pain or swelling, palpitations, or diarrhoea. He had no personal nor family history of neuromuscular disease, thyroid disease, or hypokalaemia and was not on any long-term medications or supplements.

On examination, he was afebrile but tachycardic (heart rate 116 beats per minute). Blood pressure was 127/73 mmHg and respiratory rate 16 breaths per minute. He was alert and conversant with clear speech. Tone was reduced in all four limbs, associated with absent biceps, triceps, supinator, knee, and ankle reflexes. There was profound weakness of his lower limbs and trunk (power 2/5 both proximally and distally), with less severe weakness in his upper limbs (power 3/5proximally, 4/5distally). There was no sensory deficit. Cranial nerves were intact with preservation of respiratory, ocular, and bulbar muscular function. He had a small diffuse goitre that was nontender, with no bruit. Fine tremors were present in both the hands. There were no signs of thyroid eye disease or pretibial myxoedema.

**Figure 1:**
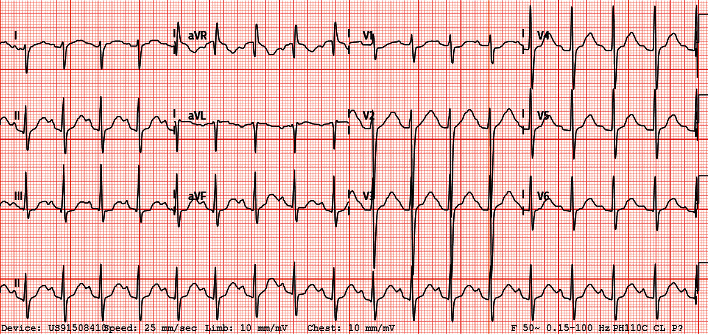
Electrocardiogram.

There was initial concern for Guillain-Barre syndrome given his acute flaccid paralysis following COVID-19 infection. Other differentials included hypokalaemic or thyrotoxic periodic paralysis (TPP). Subsequent investigations (Table 1) revealed severe hypokalaemia (1.7 mmol/L) and primary hyperthyroidism (FT4 28.96 pmol/L; TSH <0.004 mIU/L), establishing the diagnosis of thyrotoxic periodic paralysis. TSH receptor antibody was positive (2.4 U/L), consistent with Graves' disease. Electrocardiogram showed sinus tachycardia but no u-waves (Figure 1).

**Figure 2:**
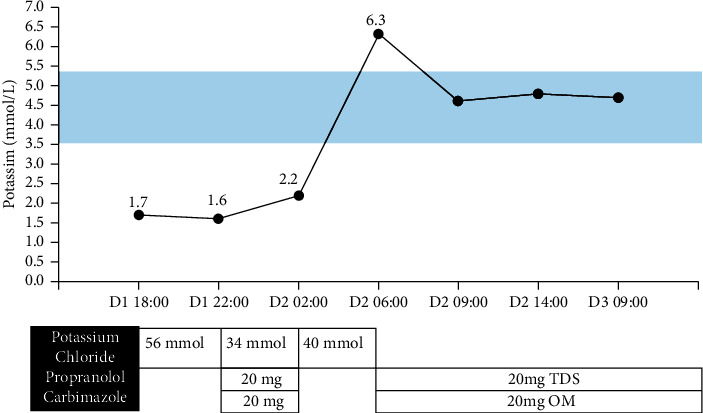
Serum potassium trend.

He was transferred to the high dependency unit, placed on telemetry monitoring, and initiated on intravenous (IV) potassium chloride (KCl) replacement. Despite receiving 56 mmol of KCl, his serum potassium level fell from 1.7 mmol/L to 1.6 mmol/L in the first four hours (prior to thyroid function test results). When primary hyperthyroidism was confirmed, he was initiated on oral propranolol 20 mg, carbimazole 20 mg, continued on IV KCl replacement, and serum potassium improved to 2.2 mmol/L. Potassium levels were checked frequently every four hours, and his subsequent potassium rose rapidly to 6.3 mmol/L. This was accompanied by prompt resolution of his paralysis, and muscle strength in all four limbs returned to normal. Potassium infusion was immediately stopped, and he was given IV Actrapid 10 u with 40 ml of 50% dextrose to correct hyperkalaemia. Serum potassium recovered to 4.6 mmol/L. Carbimazole 20 mg OM and propranolol 20 mg TDS were continued, and serum potassium levels remained stable (Figure 2). He was discharged with advice to avoid strenuous exercise and large carbohydrate portions, which may precipitate attacks of TPP [1]. He will be reviewed in a month to adjust the dosage of carbimazole, with an eventual aim for definitive therapy of Graves' disease.

## 3. Discussion

This is the first case report of TPP occurring after COVID-19 infection, which presented with acute flaccid tetraparesis. Diagnosis can be a challenge as TPP can mimic neurological complications of COVID-19 infection such as Guillain-Barre syndrome. TPP is an uncommon complication of thyrotoxicosis, rarely affecting Caucasians, and patients tend to have mild clinical and biochemical features of hyperthyroidism, making the diagnosis more difficult [2].

### 3.1. Acute Flaccid Tetraparesis Associated with COVID-19 Infection

Multiple neurological complications following COVID-19 infection have been reported, including ischaemic stroke, intracranial haemorrhage, and acute encephalitis [3]. Though rare, cases of acute myelitis and demyelinating disorders (such as Guillain-Barre syndrome) have been reported, giving rise to acute tetraparesis [4]. Postulated mechanisms include direct viral invasion of the nervous system, cytokine storm, or molecular mimicry inducing an immune response [5]. Zuberbühler et al. conducted a review of 48 published cases of Guillain-Barre syndrome associated with COVID-19 infection, with neurologic manifestations occurring at mean of 12.1 days after the onset of COVID-19 symptoms [6]. Most patients (87.5%) required treatment with IV immunoglobulin therapy.

This patient's presentation of acute tetraparesis and areflexia occurring ten days after COVID-19 infection was consistent with Guillain-Barre syndrome. However, it is imperative to exclude metabolic causes such as hypokalaemia or hypocalcaemia. Hypokalaemic and thyrotoxic periodic paralysis occur when there is an intracellular shift of potassium, leading to hyperpolarisation of cell membrane potential, and resultant flaccid paralysis and areflexia [2]. This can lead to the misdiagnosis of Guillain-Barre syndrome and emphasises the importance of obtaining results of serum electrolytes prior to instituting treatment such as immunoglobulin therapy [7].

In TTP, excess thyroid hormone mediates an increase in *β*-adrenergic receptor density and postreceptor mechanisms, which enhances tissue sensitivity to *β*-adrenergic stimulation [8]. This leads to increased sodium-potassium ATPase activity on skeletal muscles and an intracellular shift of potassium. Excess thyroid hormone also increases insulin response which in turn stimulates sodium-potassium ATPase activity [9]. TPP is a rare complication of thyrotoxicosis, with an incidence of 0.1% [10]. It is more common in Asian populations (incidence of up to 2%), and this may be due to a genetic predisposition or altered sodium-potassium channel mechanisms [11]. Like in our patient, it is most often observed in young males between 20 and 40 years of age [2].

There have not been any reported cases of TPP in association with COVID-19 infection thus far. Rajesh et al. reported a case of a 34-year-old male with COVID-19 infection who presented with hypokalaemic periodic paralysis [12]. The pathophysiology of hypokalaemic periodic paralysis appears to be similar to TPP, with shifts of potassium intracellularly being the main culprit. Association between COVID-19 and thyroiditis has been reported [13], postulated to be related to angiotensin-converting enzyme 2 (ACE-2), the receptor for cellular entry of the COVID-19 virus [14]. The mRNA encoding for the ACE-2 receptor has been found to be expressed in thyroid follicular cells, making them susceptible to viral entry, which may then give rise to thyroiditis [14]. COVID-19 infection is also known to be a trigger for various autoimmune and inflammatory diseases, mainly via molecular mimicry [15], and cases of Graves' disease occurring after COVID-19 infection have been reported [16, 17]. It is postulated that COVID-19 infection may trigger Graves' disease by altering the immune system in susceptible individuals [18]. Although this patient had a history of mild weight loss that preceded his COVID-19 infection, this had not been associated with any other symptoms of thyrotoxicosis, and the onset of paralysis occurred only after the COVID-19 infection.

Thus, this patient's recent COVID-19 infection may have exacerbated his hyperthyroidism, with his gender, age, and ethnicity making him particularly susceptible to developing TPP.

### 3.2. Treatment of Thyrotoxic Periodic Paralysis

Immediate treatment of TPP is necessary to reverse paralysis and prevent cardiopulmonary complications. For treatment of thyrotoxicosis, *β*-blocker therapy is vital in the acute phase, as it inhibits *β*-agonist stimulation and prevents intracellular shift of potassium [1]. This acts as a temporising measure till euthyroidism is achieved. Antithyroid drugs aid to control hyperthyroidism, but ultimately, these patients should be offered definitive therapy to eliminate the risk of subsequent paralysis attacks [2].

Management of severe hypokalaemia in these patients can be challenging. These patients are at risk of respiratory muscle paralysis, and fatal arrhythmias have occurred in cases of treatment delay [19–21]. Hence, immediate potassium replacement must be instituted. At the same time, caution must be exercised to avoid large quantities of potassium. Hypokalaemia in TPP is due to transcellular shift and not a total body deficit from renal or gastrointestinal losses, and reversal of the transcellular shift may lead to rebound hyperkalaemia. Hence, serum potassium levels must be monitored closely throughout replacement. Our case illustrated the difficulty in predicting the degree and rapidity of response to KCl replacement and *β*-blocker therapy. Despite propranolol and carbimazole therapy, as well as 90 mmol of KCl, patient's potassium level remained extremely low at 2.2 mmol/L with persistent severe paralysis of all limbs. However, a further 40 mmol of KCl replacement resulted in a rapid rise of serum potassium levels to 6.3 mmol/L.

A case-controlled study conducted by Lu et al. demonstrated that recovery time was significantly shorter in subjects with TPP who received IV KCl administration (10 mmol/h) compared to those who did not (6.3 ± 3.8 h vs. 13.5 ± 7.5 h) [22]. However, rebound hyperkalaemia (>5.5 mmol/L) occurred in 40% of patients who received IV KCl. Another case series of 24 patients with TPP conducted by Manoukian et al. reported that rebound hyperkalaemia (>5.0 mmol/L) occurred in 40%, especially if more than 90 mEq of KCl was given within 24 hours [23]. Thus, while KCl replacement does hasten recovery in patients with TPP, especially in those with risk of cardiopulmonary compromise, judicious amounts should be used (<10 mmol/h or <90 mmol/24 h), with continuous telemetry and close monitoring for rebound hyperkalaemia [22, 23].

## 4. Conclusion and Learning Points

COVID-19 infection has been described to trigger a myriad of complications, including various autoimmune disorders. In a patient presenting with acute flaccid paralysis following COVID-19 infection, both neurological (e.g., acute myelitis, demyelinating conditions) as well as metabolic (electrolyte disturbances and hypokalaemia) disorders should be considered. Although TPP is a rare entity, a thyroid panel should be checked in COVID-19 patients with severe hypokalaemia, bearing in mind the association between COVID-19 and Graves' disease. Last, the acute management of patients with TPP and severe hypokalaemia can be challenging, and judicious KCl replacement with frequent monitoring is imperative to avoid rebound hyperkalaemia.

## Figures and Tables

**Table 1:** Initial investigations

Test	Result		Reference
Urea	6.1	mmol/L	2.8–7.7
Sodium	141	mmol/L	135–145
Potassium	1.7 ↓	mmol/L	3.5–5.3
Chloride	106	mmol/L	96–108
Glucose	6.2	mmol/L	
Creatinine	43	Umol/L	65–125
Magnesium	0.69	mmol/L	0.65–0.95
Calcium	2.36	mmol/L	2.1–2.6
Phosphate	0.57 ↓	mmol/L	0.65–1.65
FT4	28.96 ↑	pmol/L	10–20
TSH	<0.004 ↓	mIU/L	0.4–4
FT3	17.74 ↑	pmol/L	2.5–5
TSH receptor antibody	2.4 ↑	U/L	<2
Haemoglobin	14.4	g/dL	13–17
White blood cells	13.5	×10^3^/uL	4–10
Platelets	310	×10^3^/uL	150–450
Bilirubin	13.4	Umol/L	5–30
Alkaline phosphatase	114	U/L	32–103
Gamma-glutamyl transferase	37	U/L	5–50
Alanine transaminase	41	U/L	10–55
Aspartate transaminase	24	U/L	10–45

## Data Availability

The data used to support the findings of this study are available from the corresponding author upon request.
